# 25 years of live related renal transplantation in children: The Buenos Aires experience

**DOI:** 10.4103/0970-1591.36720

**Published:** 2007

**Authors:** Eduardo Ruiz, Jorge Ferraris

**Affiliations:** Section of Pediatric Urology, Service of Pediatric Surgery, Service of Pediatric Nephrology, Department of Pediatrics, Hospital Italiano de Buenos Aires, Argentina

**Keywords:** Kidney transplantation, live related donor, pediatric

## Abstract

The number of pediatric patients with end stage renal disease (ESRD) has been steadily growing during the last 10 years all over the world, because of the improvement of medical and surgical treatment of severe urologic malformations and congenital and acquired nephrological disorders. Kidney transplantation (Tx) with a live related donor continues to be the gold standard therapy to treat ESRD in children because of the best final results, the chronic lack of cadaveric donors and the frequent possibility of young patients to have parents or relatives as a source of a potential graft donor.

Nowadays almost every pediatric patient can be dialyzed and transplanted, even early in life, if he or she has the possibility of a live related donor. Improvements in pediatric anesthesiology and intensive care have also been very important, in reducing the morbidity and mortality related to Tx procedures.

Here we report our experience with Tx for the last 25 years, specially our long experience of live related donor transplantation in children and adolescents with emphasis on technical issues in small children and pediatric patients with severe urologic malformations and bladder dysfunction. We'll make special considerations on the improvement in short and long follow-up with the actual prevention and treatment of graft rejection, due to the new immunosuppressive agents and protocols.

Kidney transplantation is the best option available to treat end stage renal disease (ESRD) in nearly every age[1–4] and is superior to dialysis replacement therapy.[5] Since 1960 kidney transplantation has been performed in children,[6] but it is only since 1980 that it has been used more frequently in small and young children with more demanding and complex pediatric, anesthesiologic and surgical requirements.[7–9] Better survival of very ill neonates with ESRD has been achieved in the last years because of dialysis techniques adapted to very small children. In spite of this improvement the number of complications and morbidity with these patients is still high.[10–11] Shortening time into a dialysis program has became an important issue in order to avoid transplant in severely ill patients, because they have no more available sites to perform any type of replacement therapy. This situation is specially true in urologic patients with many previous abdominal surgeries with the impossibility to perform peritoneal dialysis or with previous failed double lumen catheters and arteriovenous fistulas and no more sites available for hemodialysis.

Unfortunately, preemptive transplantation is not frequent in small patients because of the necessity to improve metabolic status (hyperkalemia, acidosis, uremia) and gain enough weight and space in the belly to accommodate an adult kidney. Smaller grafts coming from cadaveric pediatric donors, specially under six years of age, have not been a good option in small patients due to frequent vascular complications[12–14] though new reports on successful block transplantation, from very small donors could add a new, though rather small, pool of available kidneys for Tx.[15]

Parents and other relatives are frequently available living related donors in pediatric kidney transplantation but as intuitively expected the relation between kidney length and the available space in the retroperitoneum is out of proportion, adding extra technical difficulties. This problem is even more important in very small children due to the vascular and hemodynamic changes produced after graft revascularization.[1,16]

## GENERAL CONSIDERATIONS

Since 1981 to 2006 we have performed 235 Tx in 225 patients between one and 21 years of age (median 10.5 years); 65% patients were males, 26% patients were younger than six years (males 67.5%), average weight was 30,3 kg (8,9–79 kg). Renal insufficiency was secondary to a glomerular disease in nearly 50% of the patients due to hemolytic uremic syndrome (HUS), associated with endemic diarrhea, a severe vasculitis secondary to verocytoxicine-producing *Escherichia coli* (VTEC) as the most frequent cause of ESRD.

In Table 1 a comparison between the most frequent diagnoses shows the importance of HUS in Argentinian renal transplantation programs. Infravesical obstruction and neurogenic bladder are the most frequent urologic disorders with severe bladder involvement.

**Table 1:** Comparison of Incidence of primary renal disease in three different groups of pediatric patients with renal transplantation

Diagnosis	HIBA (1981–2006) %	Argentina (1990–2000) %	NAPRCTS 2006 %
Hemolytic uremic syndrome	24.1	18.1	2.7
Reflux and renal dysplasia	17.5	15.6	21.2
Obstructive uropathy/neurogenic bladder	19.3	25.4	18.25
Focal segmental glomeruloesclerosis	6.9	13.2	11.7
Renal cystic disease	5.1	n/a	5.7

A long follow-up is important in transplanted patients because of the possibility of recurrence of the original illness that led to ESRD, in the new kidney though as you can see in Table 1, focal segmental glomerulosclerosis (FSG) with a theoretical possibility to recidivate between 20–30% is only 6.9 % of patients. Fortunately, HUS associated with diarrhea (24,1% of our patients) has no possibility of recidivating and in fact behave postoperatively like patients with renal hypoplasia-dysplasia.

As we have only 16.8% of Tx with cadaveric donors, our historical preference is for live related donors with an immunosuppressive regimen with cyclosporine, giving us 80 and 95% graft and patient survival respectively, at five years of follow-up; fortunately, since 1999 these results have improved to reach nearly 100% for both grafts and patients with the new immunosuppressive protocol.

A new group of patients who are survivors from fetal obstructive pathology, oligoamnios and its associated lung hypoplasia has augmented the pool of patients for Tx. The possibility of very young children with ESRD to arrive to Tx has increased dramatically in the last years because of the numerous technical developments in renal replacement treatment modalities which include continuous ambulatory peritoneal dialysis (CAPD) or continuous cycling peritoneal dialysis (CCPD) even in neonates and small children.

Other factors that have improved final results in the long follow-up are the generalized use of new immunosupressive agents like specific humanized monoclonal antibodies (daclizumab), calcineurin inhibitors (tacrolimus) and inhibitors of inosine monophospate dehydrogenase (mycophenolate) that have dramatically reduced the episodes of acute rejection in the postoperative period[1,17,18] [Table 2]. Treatment and prevention of viral infections have improved patient and graft survival reducing the frequency and seriousness of complications of cytomegalovirus infection (CMV).[19,20]

**Table 2:** Changes in Immunosuppressive protocols and percentage of patients who presented episodes of acute rejection

Period/drug	Azatioprine	Prednisone	Cyclosporine	MMF	Tacrolimus	Daclizumab	Thymoglobulin*	Rejection (%)
1979–1985	X	X	O	O	O	O	O	60
1986–1995	X	X	X	O	O	O	O	40
1995–1998	O	X	X	X	O	O	O	25
1999–2006	O	X	O	X	X†	X	X	3

* Only in cadaveric Tx,

† since 2001

Nowadays Tx without previous dialysis (preemptive) is being performed more frequently with lived related donors specially in older children and adolescents because of the possibility to make an organized timing with an available donor before reaching ESRD; 24% of large series of pediatric patients have received a preemptive TX. Though cadaveric premptive Tx is theoretically possible, some Tx programs don't accept patients who are not receiving any type of dialysis. Because of this reason and the need to have a perfect timing to avoid performing Tx in a metabolically unstable patient, most of the time preemptive Tx is performed with live donors. A patient close to the time of beginning dialysis because of reduction of creatinine clearance, rising serum K or urea, poor or no somatic growth or anemia nonresponsive to erythropoietin treatment are the usual indications for dialysis or preemptive Tx specially if a live donor is ready available. Actually, very good results with this approach make it reasonable to try more frequently to do live related kidney transplantation in small and young patients without replacement therapy as has been published.[19,21,22]

Pediatric patients with low weight have not shown a higher percentage of postoperative surgical complications and final results in the short and long follow-up were similar to older patients.

Many reports in the last few years have stressed that graft survival is not adversely affected because of a reconstructed abnormal bladder, provided there is good capacity, compliance and complete emptying by spontaneous voiding or clean intermittent catheterization (CIC).[23–26] More postoperative urologic complications will be expected in this group of patients so aggressive prevention and treatment of urinary infection and reflux is mandatory.[27] We will be discussing each of these topics.

## DONOR CONSIDERATIONS

Live related donors of pediatric patients are usually young and healthy parents or relatives with a strong desire to help extremely ill children and teenagers. This concept is even more important in donors of patients who have gone through a long period of time with any type of dialysis, with the physical limitations and poor quality of life related to this replacement therapy.[9,28,29]

In spite of the nephrectomy, donors will lead a normal life after surgery and health conditions can be even better than the general population as has been published for kidney donors in the long follow-up.[30–32] Histocompatibility is not different than in adults and normally the donor is one-haplotype match.[12,18] Sibling donation with identical HLA, though possible (two patients in our group of donors) is extremely uncommon because of legal restrictions. Good results with live donor transplant are related not only to the HLA matching, but more to the reduction of postoperative acute tubular necrosis and acute rejection with an appropriate technique of transplantation and the use of a new regimen of immunosuppressive drugs.

**Figure 1:**
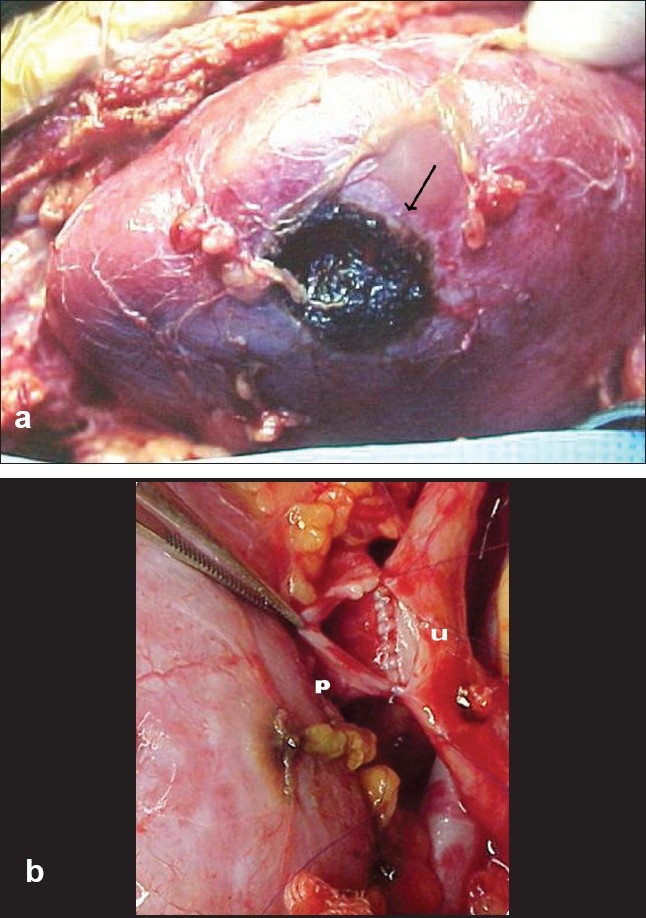
a. bench repair of a small angiomyolipoma in the donor's kidney b. pyelo (p) uretero (u) anastomosis in a donor′s kidney with UPJO

Renal sonography and urography are important to determine asymptomatic congenital (pyelocalicial duplication, hydronephrosis) and acquired (lithiasis, tumors) pathology but in our experience arteriography or magnetic resonance imaging (MRI) have been extremely useful to determine the number and position of renal arteries and choose the side of donation. Only one renal artery and single pelvicalyceal system are preferred but it is not mandatory, 10% of our patients had double arteries. Infrequent conditions in the donor kidneys like asymptomatic ureteropyelic junction obstruction (UPJO), complete pyeloureteral double system, renal ptosis or angiomyolipoma in kidneys with normal function and parenchyma are not an absolute contraindication of live donor Tx. These asymptomatic could be repaired or treated during donation procedure on the bench or even during the recipient's surgery [Figures 1a and b] with excellent postoperative results and function. This open-minded approach is useful to expand the live donor's pool.

Laparoscopic nephrectomy with lower pain postoperatively and better final cosmetic result, has been helpful to potential donors, to take a positive final decision on organ donation.

## SURGICAL CONSIDERATIONS FOR PREOPERATIVE RECIPIENT EVALUATION

Proper evaluation of abdominal great vessels is mandatory to avoid unexpected technical complications because of vena cava or iliac vein thrombosis or abnormal vessel position because of complex malformations with associated situs inversus abdominalis. This is especially true if the recipient has a previous history of catheter placement for hemodialysis in femoral veins. A thorough Doppler ultrasonography investigation of the aorta, inferior cava and iliac vessels must be performed to identify potential problems during Tx. In fact we haven't contraindicated or suspended any transplant for this reason. A special approach in such cases, is to use the upper part of the inferior cava and aorta for vascular anastomosis or more infrequently gonadal or native renal vessels of the recipient.

Peritoneal dialysis catheters are usually not a technical problem for surgery because they exit the abdomen close to the external border of the rectus fascia; so an extraperitoneal approach to the retroperitoneum is not jeopardized. Donor's kidney is harvested when possible from the side with only one renal artery and giving preference to the left side than the right. Longer length of left renal vein and possibility of transplant in right iliac fossa by turning the graft around from left to right is an advantage of the graft from the left. Implants are always performed in the right side of the recipient for being technically easier to perform venous anastomosis and position of the inferior cava. In right side donor nephrectomy, graft is turned upside down to free the renal pelvis from the lower pole so as to avoid ureteral kinking at the level of the UPJ.

There are two different types of patients with urologic malformations: with and without hydronephrosis and reflux. In the first group are patients with severe bilateral renal dysplasia with actually no reflux, previously surgically treated obstruction or reflux, survivors of Wilm's tumor and all this patient who have a normal bladder in spite of the original diagnosis. In the second group there are patients with severe non-treated vesicoureteral reflux, neurogenic bladder or infravesical obstruction like posterior urethral valves and Prune Belly Syndrome. A renal and vesical sonography, cystography and urodynamics were done in preparation for Tx to evaluate hydronephrosis, reflux and bladder and reservoir capacity, compliance and effective voiding.

## SURGICAL TECHNICAL CONSIDERATIONS

### (1) General important principles

Older children and teenagers share the same technical characteristics as an adult patient as enough space is always available into retroperitoneum to accommodate an adult kidney (9 to 12 cm in the long axis).

A wide hockey stick incision is usually performed in small patients but in older children and teenagers an oblique hypogastric incision can be performed to obtain a better postoperative cosmetic result. Lymphatic control is performed in order to avoid postoperative lymphocele during great vessels' dissection, by meticulous electrocoagulation of small lymphatics and tie sutures of the larger ones. Lymphocele is very infrequent in small patients, but must be thoroughly prevented in older patients.

Vascular anastomosis with optical magnification (3.5 or 5X) is performed to a larger vessel available looking for a short and straight passage into the retroperitoneum of the renal vein and artery. The aorta and cava have always been our preferred vessels in order to obtain the best and maximum blood flow simplifying technical details during vessels' anastomosis. Monofilament sutures 6 or 7–0 like polypropylene or Polidiaxone monofilament are preferred for this vascular procedure. Arterial anastomosis could be performed above or below the inferior mesenteric artery, but great attention must be paid to avoid damage to the mesenteric artery with the vascular clamps or sutures in small patients with very tiny vessels.

**Figure 2:**
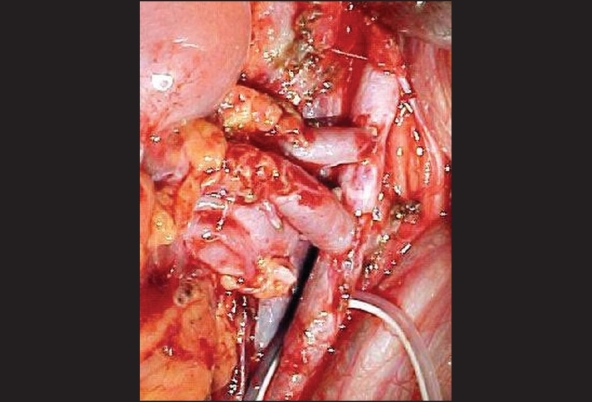
two vascular anastomosis in double renal donor′s artery to recipient's aorta

When the donor kidney had two arteries, we preferred to do separate anastomosis to the great arteries (aorta or iliac artery) [Figure 2]. A renal to renal double-barrel anastomosis on the bench is another possibility; if two veins are present, one can be ligated without vascular problems, as the venous system has wide communications.

The donor kidney is harvested in a second surgical room close to the recipient's one and is transferred to the recipient's surgical room only after all the bed and vessel dissection in the child has been completed. Donor kidney nephrectomy is completed only after transecting the ureter close to the bladder and confirming good urinary output. The donor kidney is then perfused with a cold solution of Ringer lactate with added lidocaine 1%, heparin and methylprednisone, until returning fluid is clear of blood and adequate cooling of the kidney is obtained. This procedure is normally performed through the renal artery but if the artery is too narrow to accommodate a catheter for infusion or two arteries are present, renal perfusion through the renal vein is preferred without renal impairment.

The kidney is maintained in this cold solution and on a bed of crushed ice, only separated by a plastic film as a barrier to avoid direct contact with the organ. Laparoscopic assisted donor nephrectomy has added more bench time surgery because the renal hilum is not fully prepared for Tx and the staples from the renal vein have to be removed as the first step, in order to infuse and cool the graft. As we always use the right side for transplantation and the vessels have to lie posteriorly in the retroperitoneum, we prefer to modify the ureteropyelic angle (up-down kidney) in right kidney donor to right recipient side Tx.

### (2) The very small patient

In 2006 we published a review of 23 patients with weight less than 17kg with very good results, even better than older patients, like other authors have published in the last few years.[18,33,34] Since 1985, 23 patients between one and 10 years of age (male 16 / females seven) with ESRD and a weight lower than 17kg(8,9–16,9) were operated and received their first transplantation with a live related renal donor. The etiology of the ESRD that led to kidney transplantation showed more than 50% of patients with different types of urologic problems. The results were extremely good with a graft survival at five and 10 years of follow-up of 100% and 95,6% respectively, There were no surgical complications in the short postoperative period like lymphocele or urinary leaks or stenosis in spite of the complexity of the procedures. We feel that very close observation of every surgical and anesthesiological detail are responsible for a successful outcome.

Weight 20 kg and 15 kg of the recipients has been reported in the literature as limits to perform an extraperitoneal approach.[2,35,36] In spite of these reports, we have never done a transperitoneal approach though our smaller patient's weight was 8.9 kg. In these complex cases we have performed an extensive extraperitoneal dissection, which includes wide mobilization of the retroperitoneum from the abdominal diaphragmatic side to the bladder neck. This surgical maneuver not only permits enough space for the Tx but adequate control of the iliac vessels, aorta and inferior cava preserving the possibility to do peritoneal dialysis if necessary in the postoperative period.

When an ipsilateral nephrectomy is needed, this can be performed during this stage of surgical procedure and is highly recommended in very small patients. This provides space for the graft and avoids the native kidney lying at the end of the procedure anterior to the graft, impairing subsequent biopsies or even an elective nephrectomy.

This surgical step is mandatory in Autosomal Recessive Polycystic Kidney Disease (ARPKD) and in severe hydronephrosis. When contralateral nephrectomy is needed we prefer to do it previously at an elective date, either through a retro or transperitoneal laparoscopic approach. When a ureterocystoplasty is planned simultaneously with the Tx or if this kidney's remaining function is necessary to avoid dialysis in the preoperative period (preemptive Tx), left laparoscopic nephrectomy (preserving ureter) is performed only 48h before transplantation. This surgical approach solves a complex problem in only one hospital stay, reducing the number of surgical procedures to the patients.

### (3) The dysfunctional and the augmented bladder

Many authors have published about successful renal transplantation in children and adults with abnormal bladders with different types of reconstructive surgery including bladder augmentation with stomach, intestine[23–26,37,38] and ureter.[39] In spite of this large experience, the best time for bladder augmentation in relation to Tx (after or before), is still a matter of discussion.[6,40] We have found that the exact timing of urinary tract reconstruction is determined by many factors, probably the best choice would be to have the urinary reservoir prepared before Tx but this is not always possible, dry reconstructed bladder, maintain the remaining renal function to avoid dialysis in preemptive Tx and preserve all the available ureter to do an augmentation and avoid an enterocystoplasty. Keeping in mind these concepts we have performed simultaneous reconstructive bladder surgery like ureterocystoplasty alone or combined with a Mitrofanoff continent urinary stoma for clean intermittent catheterization (CIC) during Tx. In fact, wide and tortuous ureters with massive reflux are theoretically the ideal tissue for augmentations covered because of its covered by urotelium avoiding in the long term the common complications of enterocystoplasty such as mucus, infection, lithiasis and the development of cancer. It also removing severe damaged kidney could be a prophylactic measure to avoid development of urinary infection or hypertension after Tx.

**Figure 3:**
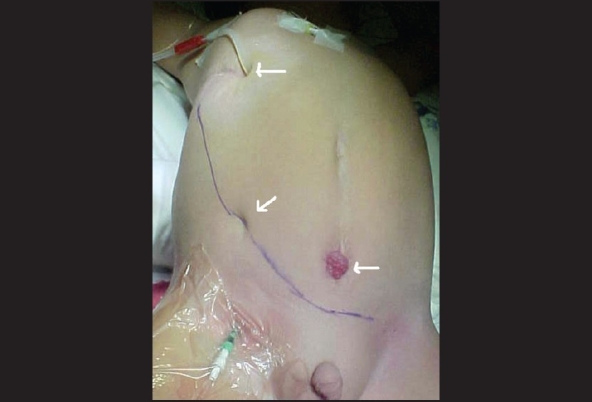
15 kg recipient, see the DPCA cannula on the right subcostal region, the vesicostomy and the wide hockey stick incision with a depression on the middle secondary to a scar because of a intrauterine vesicoamniotic shunt

We recently reviewed our experience with Tx in dysfunctional and augmented bladders; since 1996 to 2006 we have performed 29 renal Tx in 24 males and five females between one and 21 years of age (mean 11 years). The etiology of bladder dysfunction was posterior urethral valves in 11, prune belly syndrome in eight, neurogenic bladder in six and anorectal malformation in four. Previous urological procedures in preparation for Tx were a Mitrofanoff urinary stoma in 10, bladder augmentation in eight and unilateral nephrectomy in five patients. Nephrectomy in five, ureterocystoplasty in four and Mitrofanoff and orchidopexy in two were the most common simultaneous procedures. Patient and graft survival were 100% with a median follow-up of 62 months (r: 6 -135). Actual average serum creatinine is 1.3mg/dl (r: 0,4–2,3). There were no vascular complications, one moderate lymphocele resolved spontaneously and three patients required reoperation in the first postoperative week because of urological complications (ureteral necrosis, urinoma and ureterovesical stenosis). All the three patients recovered uneventfully. 46.4% of patients void spontaneously and 46.4% with clean intermittent catheterization through a Mitrofanoff stoma and one patient is still with his original vesicostomy [Figure 3].

Patients with severe vesical dysfunction had similar Tx results as urological pediatric patients with normal bladders, but required more previous, simultaneous and postoperative procedures than other pediatric patients with ESRD.[37,38,40] These multiple surgeries related to Tx help to avoid damage to the graft secondary to urinary infection and hydronephrosis. Aggressive treatment of urinary infection, obstruction and reflux in the postoperative period has been essential to avoid acquired cortical damage and maintain good graft function in the long term.

### (4) Simultaneous nephrectomy, ureterocystoplasty and Tx

If a simultaneous ureterocystoplasty is planned with both native recipient's ureters, we prefer to avoid scarring and adhesions to the retroperitoneum of this ureteral tissue with previous nephrectomy long time before Tx so we perform the left previous nephrectomy with an intraperitoneal laparoscopic procedure usually 48h before the scheduled day of Tx.

This approach permits one to free the whole left ureter from the renal pelvis to the bladder leaving right nephrectomy and right ureteral dissection to be done simultaneously with the Tx.

**Figure 4:**
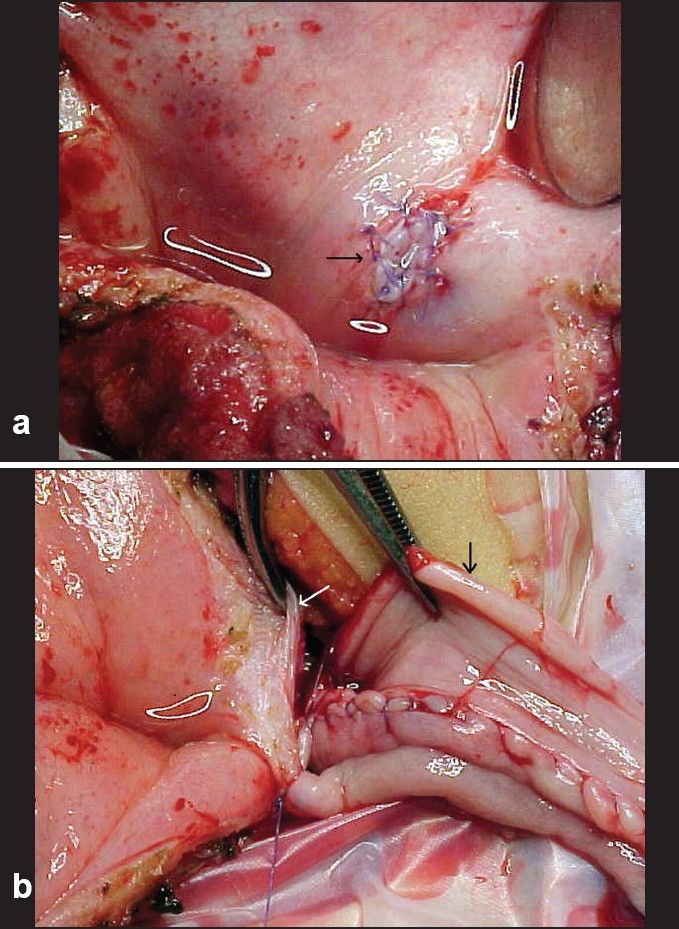
a. donor's ureter reimplanted on recipient's bladder (white arrow) b. ureterocystoplasty with all available folded ureter on the left side (black arrow)

As urine production begins as soon as vascular clamps are released, the anesthesiologist must maintain a good arterial pressure to get the best arterial flow to the Tx, by replacing one by one the lost fluids. In this way there is enough time to implant the ureter from the graft into the bladder, using the Politano-Leadbetter technique and reconstructing the lower urinary tract by finishing the ureterocystoplasty using all the available ureter tissue of the recipient [Figures 4a and b].

We have also performed simultaneous Tx and Mitrofanoff procedure in two patients by doing a small hole on the peritoneum and taking the appendix from the cecum, doing the reimplant on the left side of the available bladder mucosa using a Politano-Leadbetter-like technique and fixing the other extreme of the appendix to the skin on the umbilicus or close to it in the midline. The wide retroperitoneal dissection is an excellent opportunity to perform an orchidopexy of an intrabdominal testicle specially in a child with Prune Belly syndrome and IRCT that have not previous opportunity to solve uni or bilateral empty scrotums.

### Implanting the ureter in an augmented bladder

When the patient has a previous bladder augmentation, donor ureter reimplantation into the bladder or urinary reservoir is more difficult because of previous scars, adhesions and specially the difficulties to find a right place on the bladder muscle or intestine reservoir wall to perform the reimplantation.[2,9,40,41]

In these cases there are three technical possibilities: 1. to perform the reimplantation on the available bladder detrusor using a Lich-Gregoire technique or 2. if an implant must be done on the colonic or ileum patch a special effort must be done to construct a very long in an extravesical or intravesical approach or 3. If there is a normal ureter without reflux from the right side of the recipient a ureteroureterostomy is probably the safer approach. In every case we have always protected the urinary anastomosis with a double J catheter.

**Figure 5:**
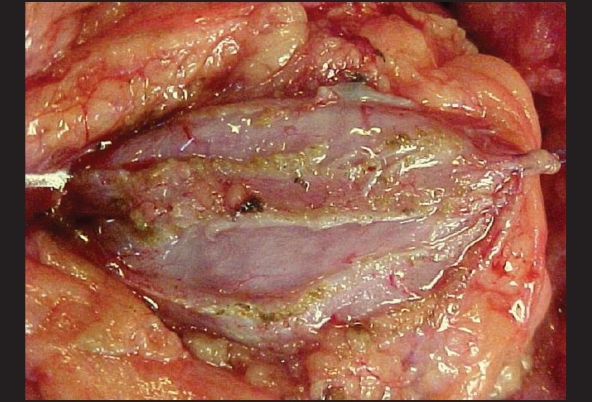
Double incision on a colonic patch of an augmented bladder, see the stripe of seromuscular tissue in the middle for improving donor ′s ureter backing

We prefer the double incision on the colon or ileum patch as seen in [Figure 5] because in this way, a steady base for the implanted ureter can be created of the whole wall of the reservoir, finally, the whole seromuscular wall can be closed over the ureter.

Bladder transitory diversion with a silicone Foley type catheter through the urethra or the Mitrofanoff stoma or even with a cystostomy must be maintained for a longer period than with a normal bladder, usually two weeks.

## IMMUNOSUPPRESSIVE PROTOCOL

Immunosuppressive protocols have been changing in the last 30 years with the aim to reduce rejection episodes and morbidity related to drugs. In Table 2 changes in the immunosuppressive regimen and the percentage of patients with rejection episodes are summarized. The very important reduction of rejection episodes in the last few years makes it difficult to compare final results between patients with different schemes of immunosuppressive drugs.

Cyclosporine was used at 5–7 mg/kg/day with the aim to maintain serum levels between 250 and 350 ng in the first postoperative month and between 50 to 100 ng in the third postoperative month and is still used by patients who received Tx between 1986 and 1998. Azathioprime is no longer used in our program, corticosteroids like methylprednisone is used at 4 mg/kg/day with progressive tapering to arrive to 0.1 mg/kg/day four months after Tx. Non free steroid regimen have been used, but very low doses (0.05 /0.08 mg/kg/day) are being tried nowadays with a selected group of patients.

Since 1993 a more selective antimetabolite, mycophenolato mofetil (MMF) replaced azathioprime with an initial dose of 1200 mg/m^2^ while receiving cyclosporine simultaneously and 600 md/m^2^ /day if the second drug is tacrolimus which has been used since 2001. Tacrolimus - a calcineurin inhibitor - replaced cyclosporine at 0.1 mg/kg/day to maintain a serum level between 5 and 10 ng/ml.[42]

Since 1999 patients for live related Tx receive a five-dose course of daclizumab 1 mg/kg/day, a humanized anti CD 25 monoclonal antibody; patients for cadaveric Tx receive a polyclonal anti-T-lymphocite antibodies (ATG) like thymoglobuline. This has been the most important factor in the reduced number of episodes of acute rejection in actual Tx in our experience and the very last reports on immunosuppressive drugs.[15,18,42,43]

In acute rejection, pulses of methylprednisolone and monoclonal antibodies like OKT3 or polyclonal antibodies like thymoglobulin have been the most used drugs.

Immunosuppressive regimen is associated with development of malignancies in the short and long term, specially PTLD (Post Transplant Lymphoproliferative Disease),[16] Two patients with live donor Tx receiving our actual scheme of rejection prevention with tacrolimus, developed PTLD in association with Epstein-Barr virus infection; both of them responded well to reduction of immunosuppressive treatment. Renal lymphoma and leukemia are other tumors which were related in our experience with cyclosporine treatment; cervical carcinoma and skin cancer are more common in transplanted patients than in the general population.[44]

## POST TRANSPLANT FOLLOW-UP

Clinical follow-up is once a week during the first three months, every two weeks between three and six months, every three weeks between six and nine months and monthly after the first year and each two and three months after the second and third year of follow-up.

While creatinine is stable and there is no urinary infection, only periodic sonographic control of the graft is required. If a surgical complication is suspected abdominal sonography and cystography are performed looking for a urinary leak or ureteral obstruction. Uro-MRI is eventually performed when results are inconclusive.

Surgical complications need prompt and aggressive treatment by an endoscopic (double J catheter), percutaneous (nephrostomy) or open surgical approach (redo ureteral reimplantation or ureteroureteroanastomosis).

Postoperative urinary infections in patients with abnormal bladder are more common than in patients with normal bladders and this fact is enhanced because of immunossupressive regimen. Though bacteriuria is extremely frequent in severe urologic patients, avoiding postoperative reflux or obstruction on the Tx kidney and maintaining effective bladder emptying are the most effective ways to avoid repeated urinary infection with subsequent renal parenchyma scars. Most of our male patients with non-neurogenic augmented bladders (posterior urethral valves and prune belly syndrome) had a urinary continent ostoma (Mitrofanoff) to avoid emptying problems secondary to pain and uncomfortable CIC through the uretha. This surgical procedure has been a useful tool to reduce urinary infection, improving bladder emptying.

Close control must be done on periodicity of CIC, especially in teenagers and adolescents when parental control is more difficult. Permanent bladder catheter drainage during the night has been extremely helpful in patients with bladder augmentation and moderate polyuria to reduce urinary infection and metabolic disturbances.[36,45] Umbilical urinary ostoma is more comfortable to do this procedure as the catheter is fixed to the abdominal wall instead of the genitalia, specially in males after puberty.

Though the subject is beyond the scope of this paper, retarded somatic growth can improve after Tx, but its cause is complex and related not only to ESRD but also to postoperative steroid treatment. Though the postoperative use of human growth hormone (rhGH) could improve growth velocity, we have a very short and small experience, particularly due to the high cost of rhGH.

## CONCLUSIONS

Renal transplantation is nowadays theoretically available for nearly all ages and original diagnosis with improved results because of the development of new technical resources and modern immunosuppressive drugs. Live related donor transplantation continues to be the gold standard treatment for pediatric ESRD especially in preemptive Tx. This approach continues to be a double responsibility for the medical treating group, because an adult wishing to donate must maintain a normal life after surgery and a very ill pediatric patient is having his great chance to grow up and improve his quality of life.

As has been mentioned in this report, strict adherence to surgical and clinical protocols for each type of patient's original illness, age and weight are extremely important to obtain the best possible results in the short and long follow-up.

While national systems are trying to obtain more cadaveric donors with good harvested and preserved grafts, these efforts will not replace, at least in the next few years, live donor Tx in children.

The results presented here must encourage pediatric urologists and nephrologists to develop local and regional programs to stimulate live donation for children and adolescents, without forgetting to work side by side with local and regional health authorities, to change the laws, improve social conditions and inform the citizens about the importance and necessity of cadaveric organ donation.
